# Supplementary material for: Unmutated IGHV1-69 CLL Clone Displays a Distinct Gene Expression Profile by a Comparative qRT-PCR Assay
Source: Biomedicines. 2022 Mar 4;10(3):604. doi: 10.3390/biomedicines10030604 (PMC8945665; doi:10.3390/biomedicines10030604)
Supplement: Supplementary file 1 [file biomedicines-10-00604-s001.zip › biomedicines-1572853-supplementary.pdf]

Supplementary Table S1. Fold expression of selected genes according to Figure 4, comparing bulk CLL1c versus bulk CLL5a and p1+ cells versus bulk CLL5a. Data are reported as folds ± SD.

Gene	CLL1c vs. CLL5a	p1+ vs CLL5a
BCL2L2	1.7 ± 0.054	4.54 ± 0.05
BCL2L1	8.7 ± 0.05	15 ± 0.06
BCL2	5.23 ± 0.03	11.76 ± 0.09
XIAP	1.4 ± 0.054	3.56 ± 0.05
ABL1	7.9 ± 0.05	7.35 ± 0.05
BAG3	6.25 ± 0.05	4.93 ± 0.05
AIFM1	0.036 ± 0.004	0.042 ± 0.004
BAD	0.52 ± 0.03	0.51 ± 0.023
DAPK1	0.02 ± 0.00345	0.02 ± 0.0034
BAX	0.13 ± 0.04	0.17 ± 0.02
HRK	0.033 ± 0.004	0.07 ± 0.004
CASP5	114.5 ± 0.0124	279,3 ± 0,12
FOS	4.5 ± 0.13	7 ± 0.13
IL1A	476.7 ± 0.67	1891.3 ± 0.67
CXCL8	36.57 ± 0.09	17.7 ± 0.09
IL1B	52.5 ± 0.45	58.93 ± 0.45
CXCL2	38.01 ± 0.31	100.97 ± 0.31
IL10	212.5 ± 0.35	877.43 ± 0.35
CCL2	115.4 ± 0.265	235.46 ± 0.26
NFKB1	13.5 ± 0.154	56.2 ± 0.15
CSF1	426.01 ± 0.45	1766.82 ± 0.45
IGF1R	1697.42 ± 0.63	3906.7 ± 0.63
CD83	1.4 ± 0.0954	6.5 ± 0.09
CD40	2.24 ± 0.065	4.98 ± 0.06
BIRC3	0.074 ± 0.0065	0.034 ± 0.006
